# The Treatment of Pneumonia

**Published:** 1907-11

**Authors:** W. T. Marrs

**Affiliations:** Peoria, Illinois


For the Texas Medical Journal.

### The Treatment of Pneumonia.

W. T. MARRS, M. D., PEORIA, ILLINOIS.

At this season of the year pneumonia articles are as thick as snow birds. Many of these are excellent, while of quite a number it may be said, like that of the serum treatment, that they are "harmless." Perhaps a few of the ideas promulgated on the subject of pneumonia treatment are capable of harm, as, for example, that of paralyzing the victim with hundred grain doses of quinine every so often. It is the consensus of medical opinion that heroic treatment is often indicated in the congestive stage of the disease, but that just referred to is going the limit.

Pneumonia is a systemic disease, with its strongest local manifestation in the lung tissue. The etiological factors are many and varied. Breathing vitiated air, exposure to dampness and general debility are fruitful causes in all ages and sexes. The absorption of toxines from the alimentary canal is a factor, if not a direct cause in nearly all cases. Too often this route for the elimination of toxic matter does not get the consideration that its importance demands. Secretion and cellular activity in general in all stages of this disease are very much below par and the phagocytic action of the blood is in a great measure overwhelmed. The bowels, liver, skin and every emunctory is dulled, if not entirely off duty. If such a condition is found, I think it is one of the few indications for big doses of calomel, say five or ten grains, until ten or twenty grains are administered. After that small doses of calomel and podophyllin should be given to maintain secretory action, one-sixth grain of each being usually all that is required. Salines are indicated throughout, for they act upon the mucous and serous coats of the intestines in a way that nothing else does. Dr. Abbott's laxative acts very nicely and has no unpleasant or nauseating effect. In case of old people who have a relaxed condition of the intestines with obstinate constipation, it is well to give an occasional colonic irrigation. In such cases antiseptics in the way of the sulphocarbolates sterilize the remaining fecal matter and thus prevent the absorption of poisons. After the patient has been made as comfortable as possible the first consideration, then, should be to keep the alimentary canal reasonably clean and sterile, with special attention also directed toward stimulation of the hepatic function.

Aconite is generally agreed to be the best circulatory sedative in the early stages of this disease, the amorphous aconitine in small doses being at present a favorite with many practitioners. It relieves nerve tension and unlocks the secretions and very strongly favors dermal elimination. Clinically atropine in small doses has a synergistic action to the aconitine, as the former causes a peripheral flow of blood, thus relieving in some measure the congested centers. Minute doses of hyoscine assist these remedies on account of its sedation, and it does not check secretion as does morphine and its congeners. The old-fashioned Dover's powder was quite generally used in pneumonia a few years ago, and many rural physicians still use it. There are yet in use many drugs that are more objectionable than Dover's powder, while we have some that should be employed in preference to it. If the patient suffers much pain codeine will be indicated. The phosphate given hypodermically in from one-half to one grain doses acts very nicely.

Carbonate of ammonia was given extensively for pneumonia several years ago, as it was thought to liquify the sputa and favor fluidity of the blood. It is a good respiratory stimulant, but is no longer used to the extent that it once was. Emetine and apomorphine are good expectorants and the writer has also employed to good advantage the old-time tartar emetic. Cough syrups would better be generally omitted. Strychnine and alcohol are our best remedies at the crisis of the disease. The arsenate of strychnine will greatly aid the patient during the convalescent stage.

In the young and rugged veratrine may be used instead of aconitine in the congestive stage along with digitaline. Unless there is a special indication for it, I do not employ strychnine at this stage of the disease.

Nothing has been said about local applications. The efficacy of the mustard plaster can not be questioned, not so much for its local effect, but because of its salutary sedative action upon certain nerve centers. Heavy and sloppy applications are harmful. In cases of delayed resolution I have blistered with the cantharidal paste and have obtained gratifying results therefrom. As to the modus operandi of its therapeusis, I am unable to say, but the test of a remedy is in the clinical results from it.

